# Supplementary material for: Clinical Outcomes of SARS-CoV-2 Breakthrough Infections in Liver Transplant Recipients during the Omicron Wave
Source: Viruses. 2023 Jan 20;15(2):297. doi: 10.3390/v15020297 (PMC9958724; doi:10.3390/v15020297)
Supplement: Supplementary file 1 [file viruses-15-00297-s001.zip › Supplementary Document S1 Questionnaire German.pdf]

UKE HAMBURG Zentrum für Innere Medizin

I. Med. Klinik und Poliklinik - Gastroenterologie, Infektiologie, Tropenmedizin
Prof. Dr. Ansgar W. Lohse
Prof. Dr. Samuel Huber
Direktoren

Ambulanzzentrum des UKE GmbH (MVZ)
Fachbereich Lebertransplantation

Universitäres Transplantations Centrum (UTC)
PD Dr. Florian Grahammer
Direktor

Ansprechpartner

Prof. Dr. Martina Sterneck
Leiterin der Studie

Prof. Dr. Marylyn Addo
Stellv. Leiterin der Studie

J. Jahnke-Triankowski
Studienkoordinatorin

Neues Klinikum O 10
Martinistraße 52
20246 Hamburg
Telefon: (040) 7410-56640
Telefax: (040) 7410-40028

Untersuchung der Immunantwort auf die Impfung mit dem adjuvantierten rekombinanten Herpes zoster-subunit Totimpfstoff (Shingrix®) bzw. auf die Impfung mit einem tetravalenten Influenza Totimpfstoff bzw. auf die Impfung gegen SARS-CoV-2 bei Patienten unter immunsuppressiver Therapie sowie mit reduzierter Immunabwehr aufgrund chronischer Erkrankungen.

Pseudonym-Code: ________________ **Datum:** ________________

Erhebungszeitpunkt: ☐ **1 Jahr** ☐ **3 Jahre** ☐ **5 Jahre**

Bitte geben Sie hier Ihre vorangegangenen Impfungen, inklusive Impfstoff und Datum an.

1.______________________________

2.______________________________

3.______________________________

4.______________________________

5.______________________________

6.______________________________

1. Wurden bei Ihnen nach einer Impfung die Antikörper gegen das SARS-CoV-2 Virus bestimmt?

☐ nein ☐ ja

2. Ist bei Ihnen inzwischen eine Covid-19 Infektion festgestellt worden?

☐ nein ☐ ja

3. Falls ja, zu welchem Zeitpunkt wurde die Erkrankung diagnostiziert?

Datum: ________________

4. Wie wurde die Erkrankung festgestellt?

☐ Abstrich mit PCR

☐ Abstrich mit Antigentest im Testzentrum

☐ Selbst durchgeführter Antigen-Schnelltest

☐ unklar

5. Kennen Sie die Virusvariante, mit der Sie infiziert waren?

☐ nein ☐ ja. Name: ____________________________

6. Wurde ein erneuter Test nach Ende der Symptome durchgeführt?

☐ nein ☐ ja

6.1 Falls ja, wann wurde der Test nach der Infektion erstmals negativ?

Datum: _____________________

6.2 Wurden Sie, nachdem Sie zunächst negativ getestet waren, im Anschluss wieder positiv getestet?

☐ nein ☐ ja

7. Haben Sie eine Vermutung, wo Sie sich angesteckt haben könnten?

☐ nein ☐ ja, Ort: ______________________________

8. Welche Symptome traten auf?

☐ Fieber
☐ Schüttelfrost
☐ Husten
☐ Schnupfen
☐ Abgeschlagenheit
☐ Halsschmerzen
☐ Geschmacksstörung
☐ Geruchsstörung
☐ Übelkeit
☐ Kopfschmerzen
☐ Durchfall
☐ Muskel-Gelenkschmerzen
☐ Kurzatmigkeit
☐ Luftnot
☐ Konzentrationsstörungen

9. Bitte geben Sie auf einer Skala von 1 (niedrig) bis 10 (sehr schwer) an, wie ausgeprägt Ihr Krankheitsgefühl insgesamt am Höhepunkt der Erkrankung war:

____

10. Wurden Sie aufgrund Ihrer Infektion medikamentös behandelt?

☐ nein ☐ ja

10.1 Falls ja, wissen Sie welche Tabletten Sie erhalten haben?

☐ nein ☐ ja. Name: ______________________________

10.2 Falls ja, haben Sie Mittel für die Symptomkontrolle, wie zum Beispiel Schmerzmittel oder Fiebersenker genommen?

☐ nein ☐ ja

10.3 Haben Sie Antikörper gegen die Virusinfektion bekommen?

☐ nein ☐ ja

10.4 Haben Sie noch andere bisher nicht genannte Medikamente bekommen?

☐ nein ☐ ja. Name: ______________________________

11. Wurde im Rahmen Ihrer Corona-Erkrankung etwas an Ihren Immunsuppressiven Medikamenten verändert?

☐ nein ☐ ja

12. Sind Sie zur Behandlung der Erkrankung im Krankenhaus gewesen? Legen Sie bitte den Arztbericht bei, falls dieser vorhanden ist.

☐ nein ☐ ja, von _______________ bis ________________

Angaben zum behandelnden Krankenhaus:

Name: ____

Abteilung: ____

Ort: ____

13. Sind während Ihrer Corona- Erkrankung erstmals Symptome aufgetreten, die über einen Zeitraum von 4 oder mehr Wochen bestehen geblieben sind?

☐ nein ☐ ja

13.1 Falls ja, welche Symptome traten auf?

☐ Fieber	☐ Halsschmerzen	☐ Durchfall
☐ Schüttelfrost	☐ Geschmacksstörung	☐ Muskel-Gelenkschmerzen
☐ Husten	☐ Geruchsstörung	☐ Kurzatmigkeit
☐ Schnupfen	☐ Übelkeit	☐ Luftnot
☐ Abgeschlagenheit	☐ Kopfschmerzen	☐ Konzentrationsstörungen
☐ Thrombose	☐ Schlaganfall	☐ Myokardinfarkt

13.2 Falls ja, bestanden diese Symptome auch über einen Zeitraum von 12 oder mehr Wochen?

☐ nein ☐ ja
