# Supplementary material for: Clinical Outcomes of SARS-CoV-2 Breakthrough Infections in Liver Transplant Recipients during the Omicron Wave
Source: Viruses. 2023 Jan 20;15(2):297. doi: 10.3390/v15020297 (PMC9958724; doi:10.3390/v15020297)
Supplement: Supplementary file 1 [file viruses-15-00297-s001.zip › Supplementary Document S2 Questionnaire.pdf]

HAMBURG Zentrum für Innere Medizin

I. Med. Klinik und Poliklinik - Gastroenterologie, Infektiologie, Tropenmedizin
Prof. Dr. Ansgar W. Lohse
Prof. Dr. Samuel Huber
Direktoren

Ambulanzzentrum des UKE GmbH (MVZ)
Fachbereich Lebertransplantation

Universitäres Transplantations Centrum (UTC)
PD Dr. Florian Grahammer
Direktor

Ansprechpartner

Prof. Dr. Martina Sterneck
Leiterin der Studie

Prof. Dr. Marylyn Addo
Stellv. Leiterin der Studie

J. Jahnke-Triankowski
Studienkoordinatorin

Neues Klinikum O 10
Martinistraße 52
20246 Hamburg
Telefon: (040) 7410-56640
Telefax: (040) 7410-40028

Assessment of vaccine immunogenicity against Herpes Zoster, Influenza or SARS-CoV-2 in patients with reduced immune response due to immunosuppressive medication or chronic comorbidities.

Pseudonym-Code: ________________ **Date:** ________________

Time of data collection: ☐ 1st year ☐ 3rd year ☐ 5 years

Please provide details of previous vaccinations, including name of vaccine and date of administration.

1.______________________________
2.______________________________
3.______________________________
4.______________________________
5.______________________________
6.______________________________

1. Was your antibody level against SARS-CoV-2 determined after the vaccination?

☐ no ☐ yes

2. Have you had a SARS-CoV-2 infection?

☐ no ☐ yes

3. If this is the case, please provide the exact date.

Date: ________________

4. How was the infection diagnosed?

☐ PCR

☐ Antigen test at an official testing site

☐ Antigen test at home

☐ unclear

5. Do you know the variant of the virus that you contracted?

☐ no ☐ yes. Name: ______________________________

6. Have you been tested again after the symptoms have resolved?

☐ no ☐ yes

6.1 If this is the case, when did you first get a negative test result?

Date: ______________________

6.2 Have you received further positive test results, after a preliminary negative test?

☐ no ☐ yes

7. Do you have a presumption where you contracted the SARS-CoV-2 virus?

☐ no ☐ yes. Location: ________________________________

8. What symptoms did you experience during your infection?

☐ Fever ☐ Sore throat ☐ Diarrhea

☐ Ague ☐ Loss of taste ☐ Muscle or joint pain

☐ Cough ☐ Loss of smell ☐ Shortness of breath

☐ Rhinorrhea ☐ Nausea ☐ Respiratory distress

☐ Fatigue ☐ Headache ☐ Concentration disorders

9. Please use a scale from 1 (mild) to 10 (severe) to provide information on the subjective severity of your illness at the peak of your illness.

____

10. Have you received medication to treat COVID-19?

☐ no ☐ yes

10.1 If so, do you know what medication you received?

☐ no ☐ yes. Name: ______________________________

10.2 If so, have you taken medication for controlling symptoms e.g., antipyretics or painkiller?

☐ no ☐ yes

10.3 Have you received treatment with monoclonal antibodies?

☐ no ☐ yes

10.4 Have you received any other medicines, not mentioned above?

☐ no ☐ yes. Name: ______________________________

11. Has your physician changed either type or dose of your immunosuppressive medication?

☐ no ☐ yes

12. Have you been hospitalized for COVID-19? If applicable, please provide the clinical report.

☐ no ☐ yes, from _______________ to ________________

Information on hospital providing treatment:

Name: ____

Ward: ____

Location: ____

13. Have you had any symptoms, that first occurred during your acute SARS-CoV-2 infection, that lasted for 4 or more weeks?

☐ no ☐ yes

13.1 If so, which symptoms persisted?

☐ Fever	☐ Sore Throat	☐ Diarrhea
☐ Ague	☐ Loss of taste	☐ Muscle or joint pain
☐ Cough	☐ Loss of smell	☐ Shortness of breath
☐ Rhinorrhea	☐ Nausea	☐ Respiratory distress
☐ Fatigue	☐ Headache	☐ Concentration disorders
☐ Thrombosis	☐ Stroke	☐ Heart attack

13.2 If so, have any of these symptoms lasted for over 12 weeks?

☐ no ☐ yes
