# Supplementary material for: Clinical Outcomes of SARS-CoV-2 Breakthrough Infections in Liver Transplant Recipients during the Omicron Wave
Source: Viruses. 2023 Jan 20;15(2):297. doi: 10.3390/v15020297 (PMC9958724; doi:10.3390/v15020297)
Supplement: Supplementary file 1 [file viruses-15-00297-s001.zip › Supplementary Table S1 LTR requiring hospitalization.pdf]

**Supplementary Table S1:** LTR requiring hospitalization due to COVID-19 related complications

| **Patient ID** | **1** | **2** | **3** | **4** | **5** | **6** | **7** | **8** | **9** | **10** | **11** |
|---|---|---|---|---|---|---|---|---|---|---|---|
| Age (years) | 68 | 72 | 69 | 56 | 67 | 71 | 72 | 62 | 65 | 65 | 65 |
| Time since transplantation (years) | 2 | 21 | 4 | 1 | 11 | 11 | 14 | 1 | 1 | 24 | 13 |
| Number of vaccine doses/ type of vaccinations | 4 Hetero-logous | 4 n/a | 4 Homo-logous | 2 Homo-logous | 3 Homo-logous | 5 Homo-logous | 3 Homo-logous | 4 Homo-logous | 4 Homo-logous | 4 Homo-logous | 3 Homo-logous |
| Interval between vaccination and infection (days) | 220 | 78 | 208 | 134 | 116 | 22 | 224 | 153 | n/a | n/a | 48 |
| Charlson comorbidity index | 10 | 14 | 6 | 4 | 14 | 9 | 10 | 7 | 8 | 7 | 10 |
| Anti-S RBD in AU/ml/ sample collection pre infection (days) | 2.7 /76 | 257.9 /33 | 1996 /75 | 10.62 /0 | 554.72 /35 | 572.7/87 | 0.4 /32 | 508.3 /9 | 1726 /69 | 1872 /48 | 0.4 /0 |
| Main reason for hospitalization | AKI due to enteritis | Pneu-monia | AKI | Pneu-monia | Pneu-monia | Pneu-monia and AKI | Pneu-monia | Somno-lence and AKI | Syncope and AKI | Pneu-monia | Pneum-onia and AKI |
| NIH disease severity | n/a | Severe | n/a | Moderate | Severe | n/a | n/a | Critical | n/a | Critical | Severe |
| ICU Admission | No | No | No | No | No | No | No | Yes | No | Yes | No |
| Pneumonia | No | Yes | No | Yes | Yes | Yes | Yes | Yes | No | Yes | Yes |

| | | | | | | | | | | | |
|---|---|---|---|---|---|---|---|---|---|---|---|
| Pulmonary infiltrates | No | Yes (CXR) | Yes (CXR) | Yes (CXR) | Yes (CT) | Yes (CXR) | Yes (CXR) | Yes (CT) | No | Yes (CXR) | Yes (CT) |
| Supplemental oxygen | No | Yes | No | No | Yes | Yes | Yes | Yes | No | Yes | Yes |
| $SpO_2$ (%) | 94 | 90 | n/a | 94 | 80 | n/a | n/a | 92 | 95 | 75.1 | 89 |
| Acute Kidney Injury † | Yes (3) | No (-) | Yes (2) | No (-) | Yes (3) | Yes (1) | No (-) | Yes (3) | Yes (1) | No (-) | Yes (3) |
| Secondary Infection | No | Yes | No | Yes | Yes | Yes | Yes | Yes | No | Yes | Yes |
| Hospitalization within 5 days of infection onset | No | n/a | No | Yes | No | No | Yes | No | Yes | No | No |
| Length of hospital stay | 7 | 23 | 9 | 7 | 12 | 3 | 9 | 120 | 4 | 19 | 39 |
| COVID-19 specific therapy | Tixagevimab/Cilgavimab | Dexamethasone | None ‡ | Sotro-vimab | None | None | Dexa-metha-sone, Sotrovimab | Rem-desivir | Rem-desivir | Dexa-metha-sone | Rem-desivir, Dexa-metha-sone |

† KDIGO classification is given in parenthesis

‡ Participants that did not receive COVID- specific therapy where hospitalized late within the disease course (>5 days post onset of infection)

<u>Abbreviations</u>

LTR Liver transplant recipients

COVID-19 coronavirus disease 2019

n/a Not available

anti-S RBD anti-SARS-CoV-2 receptor-binding domain

| | |
|---|---|
| AU | Arbitrary units |
| AKI | Acute kidney injury |
| NIH | National Institutes of Health |
| ICU | Intensive Care Unit |
| CXR | Chest X-ray |
| CT | Computer tomography |

Table Legend

Characteristics and disease course of LTR requiring hospitalization due to COVID-19 related complications.
