# Supplementary material for: Clinical Outcomes of SARS-CoV-2 Breakthrough Infections in Liver Transplant Recipients during the Omicron Wave
Source: Viruses. 2023 Jan 20;15(2):297. doi: 10.3390/v15020297 (PMC9958724; doi:10.3390/v15020297)
Supplement: Supplementary file 1 [file viruses-15-00297-s001.zip › Supplementary Table S2 LTR not requiring hospitalization.pdf]

Supplementary Table S2: LTR hospitalized for non-COVID-19-related complications or surveillance

**Patient ID**	**1**	**2**	**3**	**4**	**5**	**6**	**7**	**8**	**9**	**10**
Age (years)	42	43	40	38	50	36	85	71	29	40
Time since transplant-ation (years)	1	3	1	5	11	9	24	9	18	1
Vaccine doses	4	4	3	3	3	3	4	3	4	3
Interval between vaccination and infection (days)	21	204	427	125	105	217	n/a	178	n/a	n/a
Charlson comorbidity index	3	5	3	3	4	5	8	5	3	9
Anti-S RBD in AU/ml/ sample collection pre infection (days)	3666 /0	6.27 /30	44.84 /24	10,507 /100	n/a	n/a	1703 /0	n/a	50.88 /0	48.52 /14
Main reason for hospitali-zation	Assess-ment in ER	Pares-thesia	Graft dys-function	Assess-ment in ER	Excision of Basal Cell Car-cinoma	Chol-angitis	Bloody Diarrhea	Clinical monitoring of COVID-19	Assess-ment in ER	Chol-angitis lenta
COVID-19 symptoms	Sore Throat, Fever	Asymptom atic	Sore Throat, Fatigue, Myalgia	Sore Throat, Cough, Fatigue	Fever, Cough, Fatigue	Asympto-matic	Asympto-matic	Fever	Rhino-rrhea	Cough

Nosocomial infection	No	No	Yes	No	No	No	No	No	Yes	Yes
Length of hospital stay (days)	2	5	7	1	4	9	7	2	1	45
COVID-19 specific therapy †	None	Tixa-gevimab/ Cilga-vimab, Rem-desivir, Sotro-vimab	Rem-desivir, Sotro-vimab	Molnu-piravir	None	None	None	None	Rem-desivir, Tixa-gevimab/ Cilga-vimab	Rem-desivir, Sotro-vimab

† Administration of COVID-specific treatment was based on availability of drugs and individual factors, such as age and comorbidities, antibody level, symptoms, and time of admission during disease course

LTR	Liver transplant recipients
COVID-19	coronavirus disease 2019
n/a	not available
Anti-S RBD	anti-SARS-CoV-2 receptor-binding domain
AU	Arbitrary units
ER	Emergency Room

Table Legend

Characteristics, reason for hospitalization and course of SARS-CoV-2 infection in liver transplant recipients (LTR) not requiring hospitalization.
