# Supplementary material for: Clinical Outcomes of SARS-CoV-2 Breakthrough Infections in Liver Transplant Recipients during the Omicron Wave
Source: Viruses. 2023 Jan 20;15(2):297. doi: 10.3390/v15020297 (PMC9958724; doi:10.3390/v15020297)
Supplement: Supplementary file 1 [file viruses-15-00297-s001.zip › Supplementary Table S3 LTR with prolonged positivity.pdf]

Supplementary Table S3: LTR with prolonged SARS-CoV-2 positivity

**Characteristics**	**LTR without prolonged SARS-CoV-2 positivity n=90**<br>n (%) / median (IQR)	**LTR with prolonged SARS-CoV-2 positivity n=8**<br>n (%) / median (IQR)	**p-value**
Age at time of infection (years)	56 (42-65)	58.5 (32.8-67.3)	0.982
Females	43 (47.8)	3 (37.5)	0.719
Time since transplantation (years)	7 (3-14)	5.5 (1-10.5)	0.385
**Risk factors**			
Diabetes	28 (31.1)	4 (50)	0.432
Arterial Hypertension	48 (53.3)	4 (50)	1.0
Age >60 years	33 (36.7)	4 (50)	0.471
eGFR <30 ml/min	16 (17.8)	2 (25)	0.639
BMI >30 kg/m^2	12 (13.3)	1 (12.5)	1.0
2 risk factors	42 (46.7)	4 (50)	1.0
≥3 risk factors	18 (29)	3 (37.5)	0.363
Charlson comorbidity index	5 (4-8)	6 (4-9.75)	0.496
**Vaccination status**			
Second dose	9 (10)	3 (37.5)	0.056
Third dose	57 (63.3)	2 (25)	0.056
Fourth dose	23 (25.6)	2 (25)	1.0
Fifth dose	1 (1.1)	1 (12.5)	0.157
Received Booster dose	81 (90)	5 (62.5)	0.056
**Immuno-suppression**			
Mono	21 (23.3)	1 (12.5)	0.679
CNI + MMF/mTORi/ Prednisone/Azathioprine	50 (55.6)	5 (62.5)	1.0
mTORi + MMF/Prednisone	3 (3.4)	2 (25)	0.052
≥3 Immunosuppressants	16 (17.8)	0	0.346

Including MMF	27 (30)	5 (62.5)	0.109

Comparison of LTR with known prolonged SARS-CoV-2 positivity and those without. Frequencies and percentages (n= (%)) are given for nominal and ordinal variables. For numerical variables median and interquartile range (median (IQR)) were calculated. Statistical analysis was performed with Pearson's chi-squared Test, Fisher's exact test or Mann-Whitney-U test.
