# Supplementary figures and images for: Clinical Outcomes of SARS-CoV-2 Breakthrough Infections in Liver Transplant Recipients during the Omicron Wave
Source: Viruses. 2023 Jan 20;15(2):297. doi: 10.3390/v15020297 (PMC9958724; doi:10.3390/v15020297)

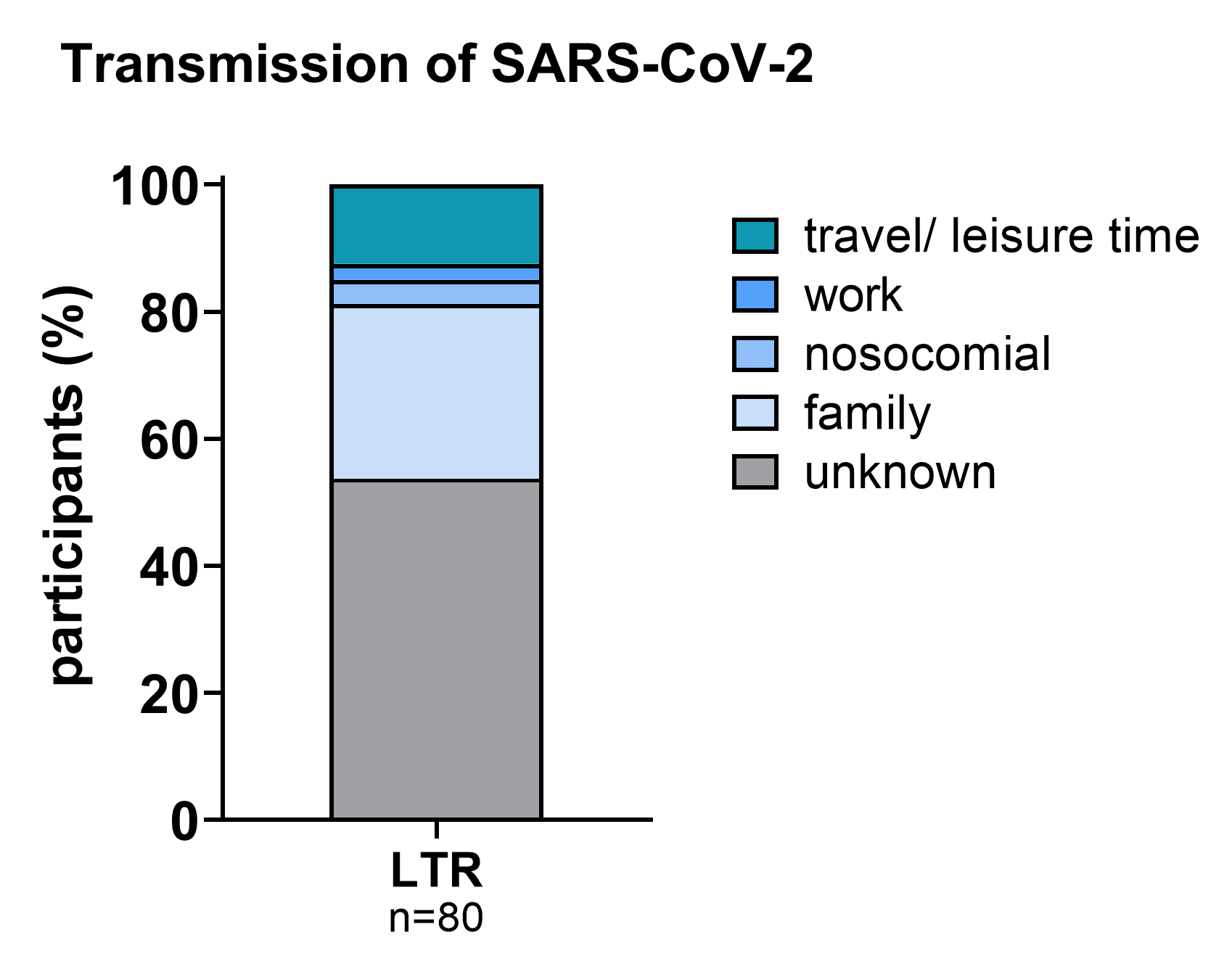

Supplement: Supplementary file 1 [file viruses-15-00297-s001.zip › Supplementary Figure S1.tif]

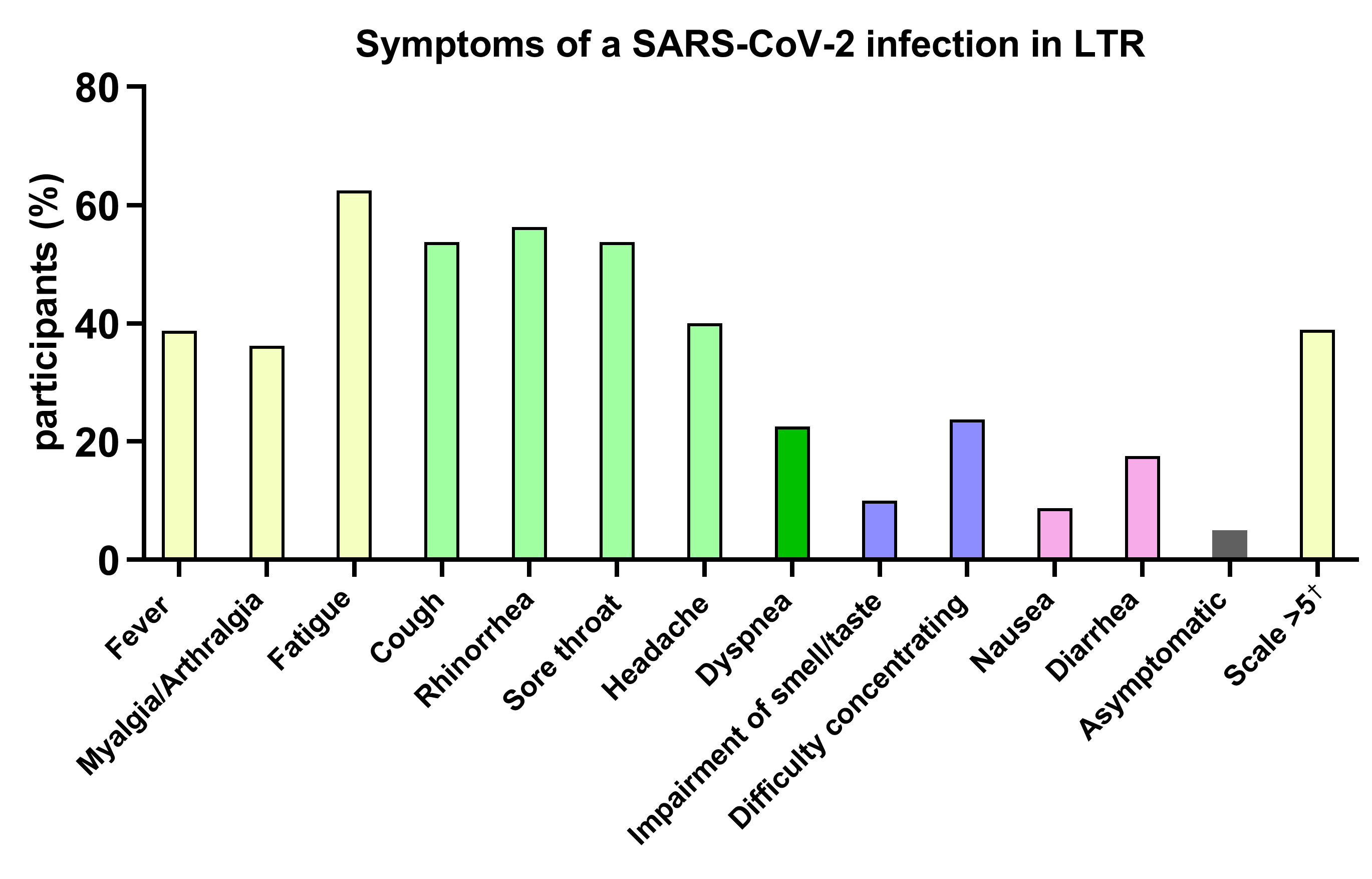

Supplement: Supplementary file 1 [file viruses-15-00297-s001.zip › Supplementary Figure S2.tif]

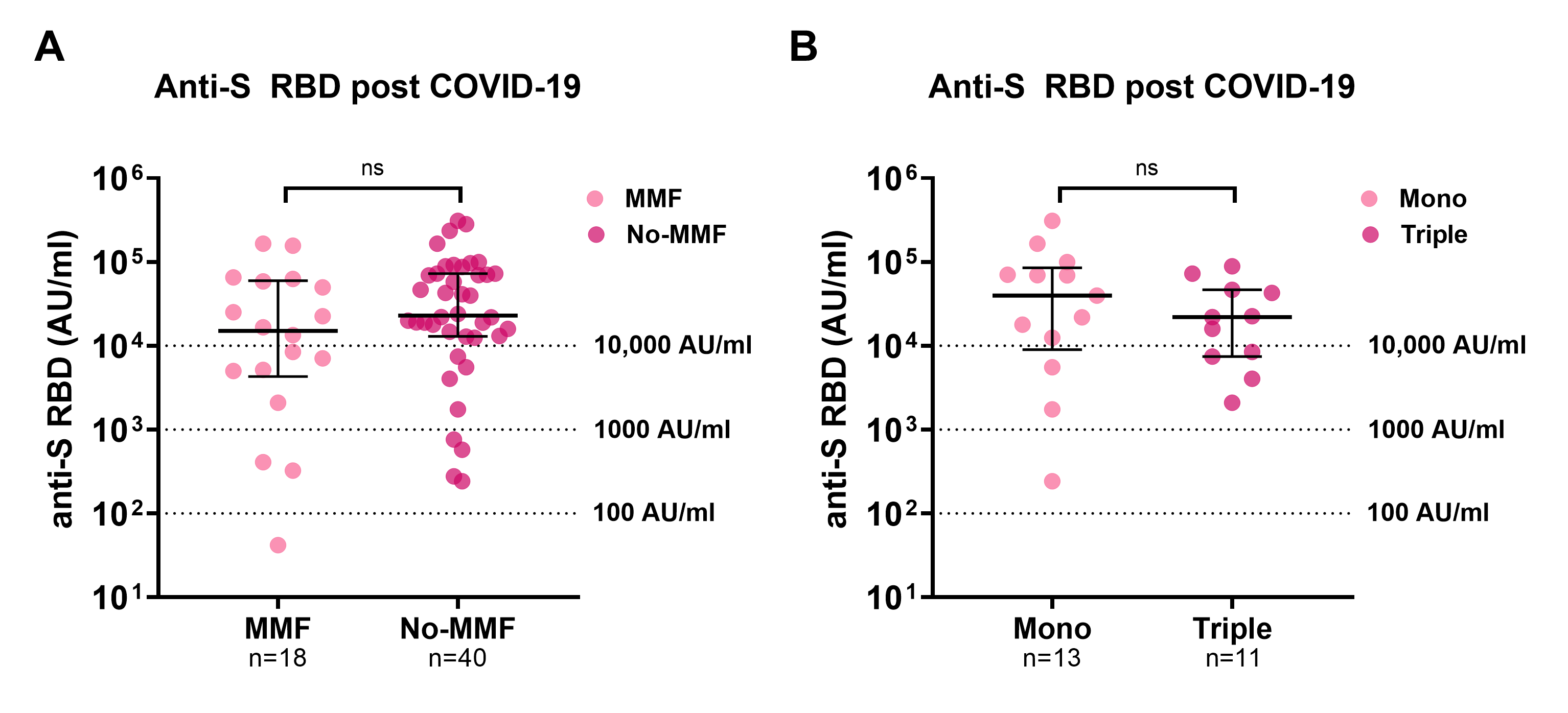

Supplement: Supplementary file 1 [file viruses-15-00297-s001.zip › Supplementary Figure S3.tif]

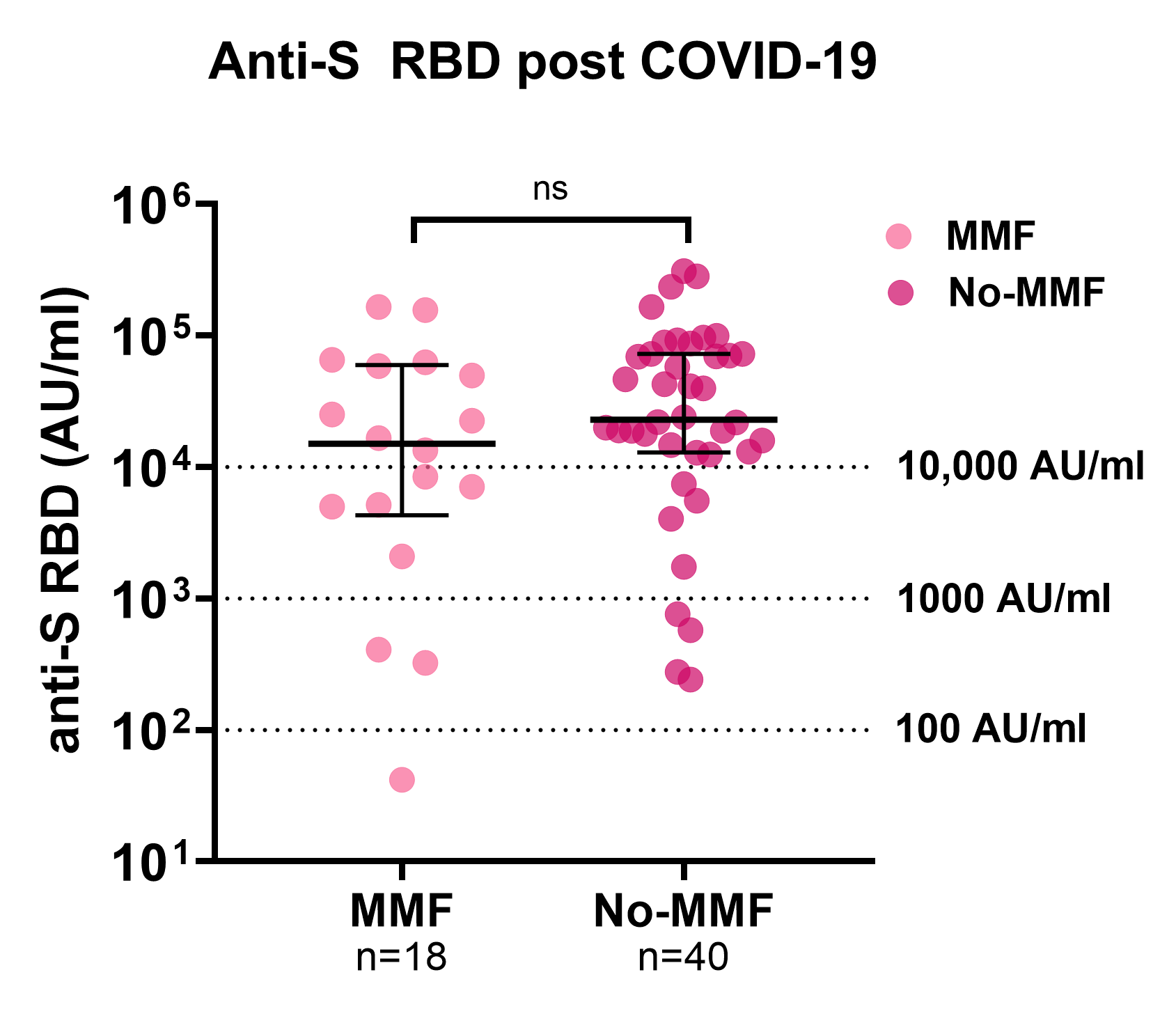

Supplement: Supplementary file 1 [file viruses-15-00297-s001.zip › Supplementary Figure S3A.tif]

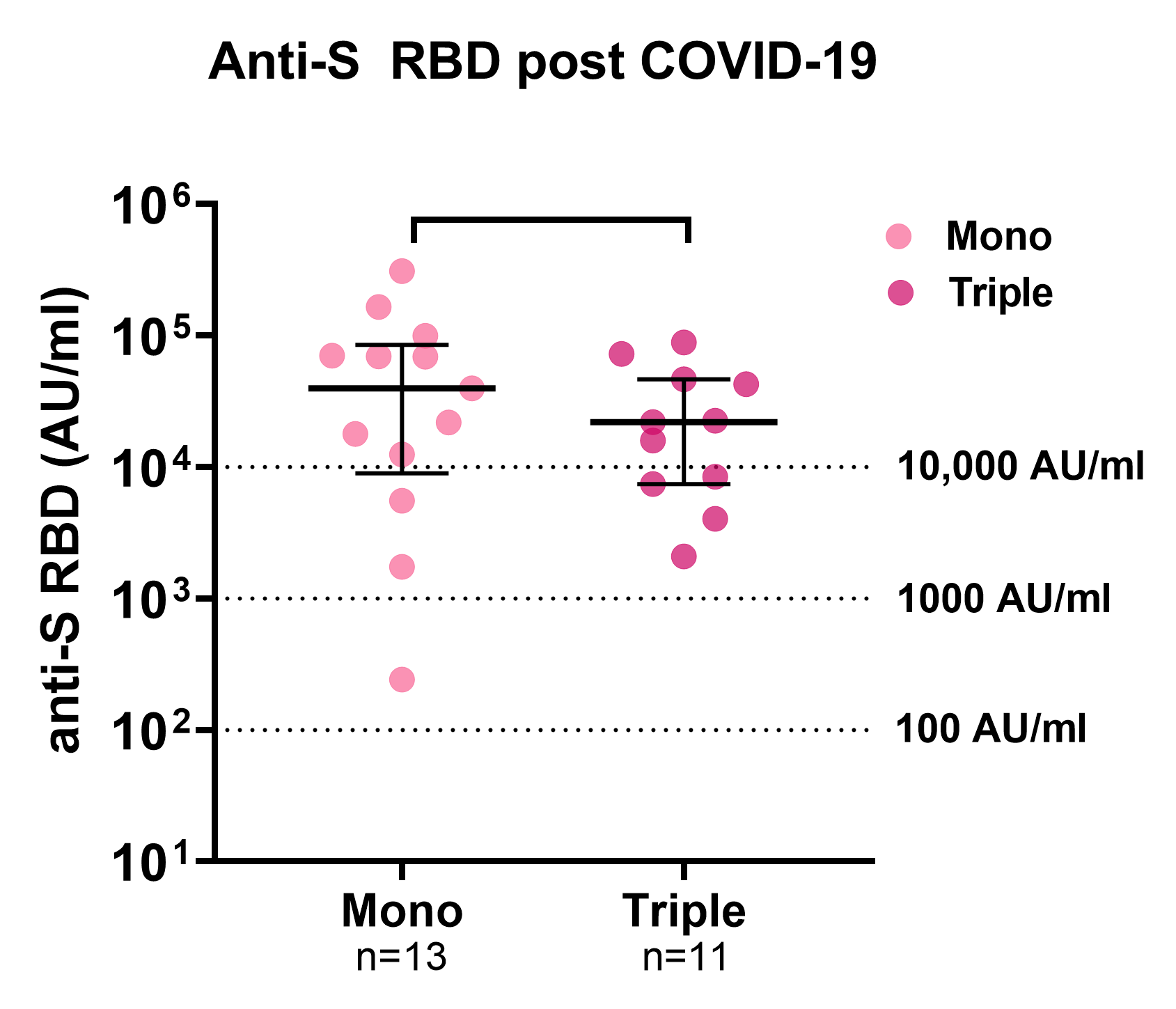

Supplement: Supplementary file 1 [file viruses-15-00297-s001.zip › Supplementary Figure S3B.tif]
